# Utility of the Electrocardiogram in Drug Overdose and Poisoning: Theoretical Considerations and Clinical Implications

**DOI:** 10.2174/157340312801784961

**Published:** 2012-05

**Authors:** Christopher Yates, Alex F Manini

**Affiliations:** 1 Attending Physician, Emergency Medicine Department / Clinical Toxicology Unit, Hospital Universitari Son Espases, Palma de Mallorca, Spain; 2 Assistant Professor of Emergency Medicine Division of Medical Toxicology Mount Sinai School of Medicine Elmhurst Hospital Center New York, NY

**Keywords:** Toxicology, electrocardiography, poisoning.

## Abstract

The ECG is a rapidly available clinical tool that can help clinicians manage poisoned patients. Specific myocardial effects of cardiotoxic drugs have well-described electrocardiographic manifestations. In the practice of clinical toxicology, classic ECG changes may hint at blockade of ion channels, alterations of adrenergic tone, or dysfunctional metabolic activity of the myocardium. This review will offer a structured approach to ECG interpretation in poisoned patients with a focus on clinical implications and ECG-based management recommendations in the initial evaluation of patients with acute cardiotoxicity.

## INTRODUCTION

Emergency physicians are frequently confronted with drug overdose. Poisoning, defined as exposure to any drug, chemical, or toxin that results in injury, is the second leading cause of injury-related fatality in the United States of America (USA) behind only motor-vehicle collisions [1]. Cardiotoxicity from poisoning is one of the leading causes of death among these patients [2,3]. It is thus crucial for clinicians to rapidly recognize cardiotoxicity and be prepared to face management decisions that require prompt action despite access to limited clinical data.

Although poisoning is an infrequent cause of cardiac arrest in elderly patients, it is the leading cause of cardiac arrest in patients under 40 years of age [1-4]. There are over two million suspected acute poisonings reported to Poison Control Centers in the USA each year [2]. Many recommendations for the emergency cardiovascular care of poisoned patients are based on expert consensus, not scientific evidence [4] though some toxin-specific recommendations for life support measures based on limited scientific evidence have been made [5]. Additionally, because standard guidelines for emergency cardiovascular care may not be optimal for the management of acute poisoning and overdose, urgent consultation with a medical toxicologist or regional Poison Control Center is recommended for patients with cardiovascular toxicity by the American Heart Association, the American Academy of Clinical Toxicologists, and the American College of Emergency Physicians [4, 6, 7]. According to recently published guidelines from the American Heart Association, the emergency cardiovascular care of myocardial injury should change in both diagnostic evaluation and therapeutic management if the patient has a history of drug or toxin exposure [8]. 

## MANAGEMENT OF POISONED PATIENTS: SIGNS, SYMPTOMS, AND ECGS

Clinicians are faced with many unknowns when managing poisoning or suspected overdose. The initial evaluation of the poisoned patient is based on the constellation of vital signs, symptoms and signs on physical examination, which may be a daunting task especially without formal training in toxicology or when faced with a rare exposure. Care of the patient takes on another element of complexity when one considers that toxicity may change during the course of evaluation depending on the particular exposure. For example, a patient with tricyclic antidepressant toxicity may undergo several phases of multi-organ toxicity prior to ultimate cardiovascular collapse. Thus, the identification of any possible toxic syndrome, or “toxidrome,” is considered the key to the initial management of the poisoned patient. Toxidrome identification, rather than focusing on toxins or suspected toxins, allows for a more rational approach to the poisoned patient. Including ECG interpretation in the initial approach can provide key information to guide management.

This review describes the role of ECG in poisoning, summarizes specific toxic effects on the myocardium and provides a systematic interpretation of the ECG and the identification of ECG toxidromes. Management issues in those patients identified to suffer from cardiotoxic effects are also addressed.

## ROLE OF THE ECG IN POISONING

Due to its widespread use, accessibility, low cost and non-invasive nature, electrocardiography is an invaluable tool in many specialties of medicine. One of its most attractive characteristics for emergency medicine and critical care is that electrocardiogram (ECG) results are rapidly available in a matter of minutes. The ECG represents the output of cardiac electrical activity detected by electrodes on the patient’s skin, which is processed by filtering and amplification to display the net result of this activity over the course of time. The waveforms and intervals produced by the electrical forces of depolarization and repolarization and their behavior over time enable physicians to identity normal and abnormal patterns that may represent cardiac or extracardiac disturbances. 

In medical toxicology, the ECG plays an important role in the evaluation of poisoning to identify or exclude cardiotoxicity, as well as to take fundamental steps in initial management. A sound understanding of ECG interpretation and the characteristics of cardiotoxicity is necessary to establish a basis for the utility of the ECG in drug overdose. A recent study found that the initial interpretation of ECGs reported to a poison center was frequently inaccurate and suggests that correct interpretation would imply changes in management recommendations [9]. Therefore, treatment implications including consultation with medical toxicologists or Poison Control Centers should be based on correct interpretation in order to lay a sound foundation for management.

ECG interpretation is well established in patients with chief complaints of chest pain and dyspnea and in those with metabolic disturbances. In the poisoned patient, the same systematic approach to the ECG can also be useful. Poisoned patients can present ECG changes common in coronary artery disease, electrolyte abnormalities or causes of dyspnea. This can be illustrated by the example of a cocaine user presenting with chest pain and dyspnea, since consequences of cocaine use may include myocardial infarction, pulmonary embolism or pneumothorax [10]. A patient’s ECG may also be scrutinized to detect changes suggestive of ion disturbances such as hypo- or hyperkalemia, hypomagnesemia or hypocalcemia; all of which may be present as a consequence of, or as an exacerbating factor in poisoning. 

Some caveats should be taken into account when analyzing the role of the ECG in poisoned patients. Factors besides direct cardiotoxicity may influence ECG changes. Severely poisoned patients with substances that do not have specific cardiac effects can present with cardiotoxicity in the context of multiorgan failure. Sympathomimetic and anticholinergic effects are commonly seen in drug overdose as well as hypovolemia and hyperthermia, all of which can cause tachycardia. Anxiety and pain may also be exacerbating factors. A patient’s baseline ECG, if known, may be useful to evaluate whether suspected acute changes are in fact present on a prior ECG.

Repeated ECG evaluation should be performed in patients with suspected cardiotoxicity or in those with ECG changes suggestive of toxicity. Patients that have ingested sustained release preparations of suspected cardiotoxic drugs or those who have ingested drugs with known delayed toxicity should also undergo serial ECGs. All such patients should be continuously monitored in an appropriate setting until toxicity resolves. 

## SPECIFIC TOXIC EFFECTS ON MYOCARDIUM

Drug cardiotoxicity may reflect disturbances on the delicate balance of the myocardial membrane potential. Cardiotoxic agents have effects on specific ion-channels (particularly sodium, calcium, and potassium) that produce important changes in the action potential as well as resting potential. What follows will be a brief review of the primary mechanisms of toxins on the myocardial ion channels and the subsequent consequences that lay the basis for ECG interpretation in poisoned patients.

### Sodium Channel Blockade

**Fig. (1):**
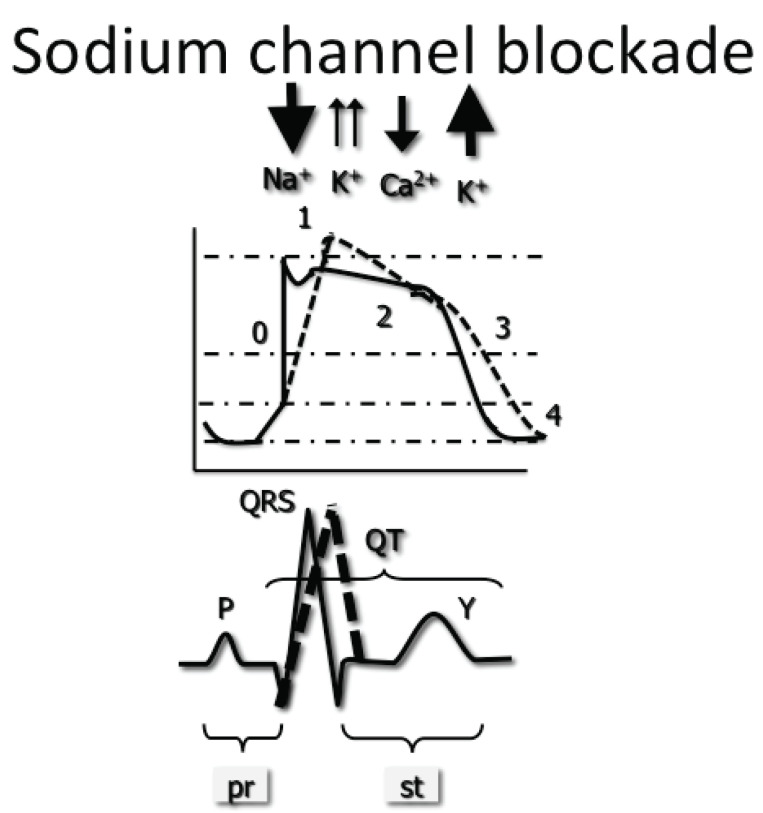
Blockade of the sodium channel leads to a delay in the fast influx of sodium. This is represented by a less pronounced slope of phase 0, normally almost entirely vertical. The greater the degree of blockade, the wider the resulting QRS complex.

The upward stroke of the myocardial action potential is a result of the conformational change and opening of voltage dependent sodium channels in response to an electrical stimulus from adjacent cells, thus causing depolarization. This phase 0 of the action potential is delayed, demonstrating a less steep slope of depolarization (Fig. **1**). There are several ECG changes suggestive of delayed ventricular depolarization. QRS morphology, duration and axis changes due to sodium channel blockade will be described in the interpretation of the ECG in poisoning. 

### Potassium Efflux Blockade

**Fig. (2):**
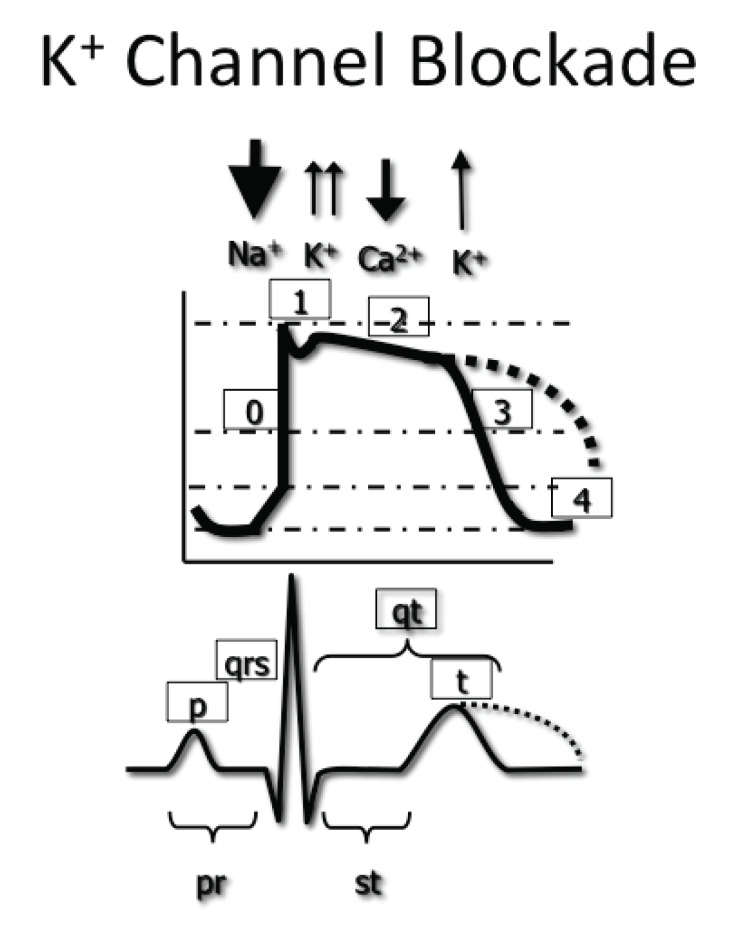
Delayed potassium efflux prolongs the action potential repolarization (dotted line) and is represented in the ECG as a prolonged QT interval.

**Fig. (3):**
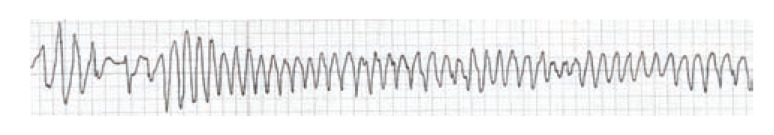
A rhythm strip showing Torsade de Pointes: a rapid polymorphic tachycardia with characteristic "twisting" of the QRS complexes around the baseline.

Myocardial membrane permeability to potassium efflux is responsible for repolarization, or the return to myocyte resting membrane potential. Potassium channel blockade that interrupts the rectifying potassium current produces an increased duration of phase 2 and phase 3 of the myocardial action potential and translates to the ECG primarily as a prolonged QT interval (Fig. **2**). Blockade of the rectifying potassium channels may also cause T-wave abnormalities or the presence of U-waves. The presence of a long QT represents slowed repolarization, which produces the myocardial substrate for the development of polymorphic ventricular tachycardia, or *torsades de pointes *(TdP) (see Fig. **3**). 

### Digoxin and Other Cardioactive Steroid Toxicity

In therapeutic doses, cardioactive steroids influence electrolyte homeostasis during phase 4 of the action potential through inhibition of the Na/K ATPase exchanger. The subsequent increased intracellular calcium concentrations are responsible for digoxin’s positive inotropic effects. The action of cardioactive steroids on the vagus nerve also produces direct suppression of impulses from the sinoatrial (SA) and atrioventricular (AV) nodes. 

### Beta Blockade

Beta adrenergic antagonism produces inhibition of catecholamine effects through competitive inhibition of Beta adrenergic receptors. Beta adrenergic stimulation produces a cAMP regulated increase in intracellular calcium. The antagonism of these receptors in the myocardium, primarily Beta-1, produces decreased automaticity, negative chronotropy and inotropy. 

### Calcium Channel Blockade

Agents that antagonize L-type voltage dependent calcium channels produce decreased entry of calcium into the cardiac cell and myocardial depression. Calcium channel antagonism at the SA and AV nodes produce bradycardia and conduction blocks due to the impairment of slow calcium channels in these tissues. The resulting direct effects are bradycardia, AV conduction disturbances that can reach complete heart block and myocardial depression. If peripheral hypotensive effects prevail, reflex tachycardia can result. 

## HOW TO APPROACH THE ECG IN THE POISONED PATIENT

### Systematic Interpretation 

Adapting a systematic method to ECG interpretation in poisoned patients can help clinicians avoid missing key signs of drug cardiotoxicity. The approach to the ECG should include evaluation of the rate, rhythm, intervals, wave morphologies, and careful attention to evidence of ischemia or infarction. In addition, some elements of cardiac injury specific to toxicology may also be present on the ECG. 

### Rhythm – Rate

In most patients, and particularly in unstable patients, initial clues to drug cardiotoxicity will be discovered when analyzing rhythm. The origin of the rhythm (supraventricular, ventricular) and especially the existence of bradycardia with or without AV-block and tachycardia (with narrow or wide ventricular complexes) is pertinent to toxicologic analysis of the ECG. When analyzing rhythm, the presence of ectopy should be noted and may represent enhanced automaticity (e.g. cardioactive steroid toxicity, sympathomimetics) or severe electrolyte disturbances. Life threatening dysrhythmias such as ventricular tachycardia, ventricular fibrillation and complete AV-block should be addressed immediately according to Advanced Cardiac Life Support (ACLS) guidelines, however the emergency cardiovascular care of myocardial injury should change in both diagnostic evaluation and therapeutic management if the patient has a history of drug or toxin exposure [8]. Some issues regarding toxicology-specific ECG findings are addressed below. 

### PR Interval

**Fig. (4):**
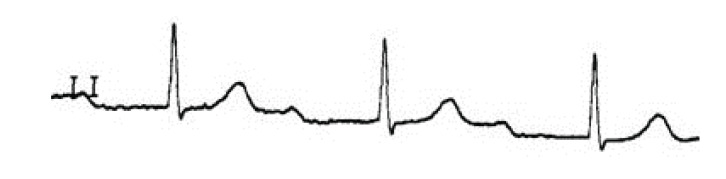
Prolonged PR interval in first-degree AV block.

**Fig. (5):**
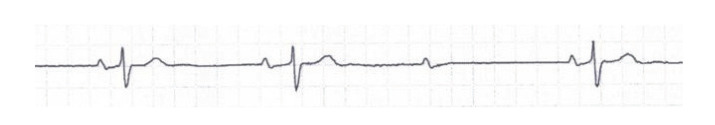
In Type 1 second-degree AV-block an increasing PR interval precedes a blocked atrial beat.

**Fig. (6):**
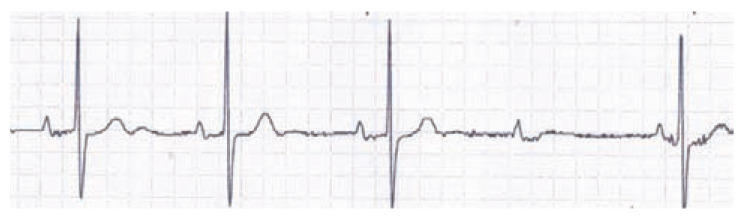
A constant PR interval preceding missed atrial beats in Type 2 Second degree AV-block.

**Fig. (7):**
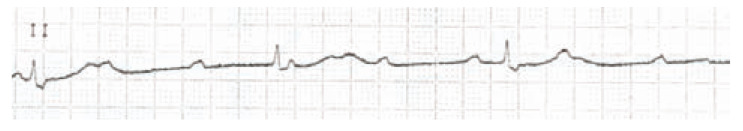
This ECG demonstrates independent atrial and ventricular activity characteristic of third degree AV-block with a bradycardic ventricular escape rhythm.

A prolonged PR interval (Fig. **4**) can be an early sign of Beta adrenergic antagonism, calcium channel antagonism, or cardioactive steroid (e.g. digoxin) effect. However, other substances that may rarely decrease sympathomimetic tone or increase vagal tone include opioids, clonidine or sedative-hypnotics [11, 12]. Scrutiny of the PR interval can identify rhythm disturbances other than first degree AV-block. An increasing PR interval preceding blocks defines type 1 second degree AV-block (Fig. **5**) whereas intermittently conducting atrial beats without a preceding PR prolongation and posterior shortened PR are present in type 2 second-degree AV block (Fig. **6**). Independent atrial and ventricular activity is characteristic of third-degree AV-block (Fig. **7**). 

### QRS Interval

Manifestations of sodium channel blockade can be found in the duration or the axis of the QRS complex. Most of the literature analyzing QRS prolongation due to sodium channel blockade comes from the experience with tricyclic antidepressant poisoning. However, other drugs that produce sodium channel blockade (e.g. Class IA antidysrhythmics) can cause prolonged QRS duration, with or without other ECG findings characteristic of TCA overdose. Table **1** summarizes common drug classes that produce sodium channel blockade and QRS prolongation. 

**Fig. (8):**
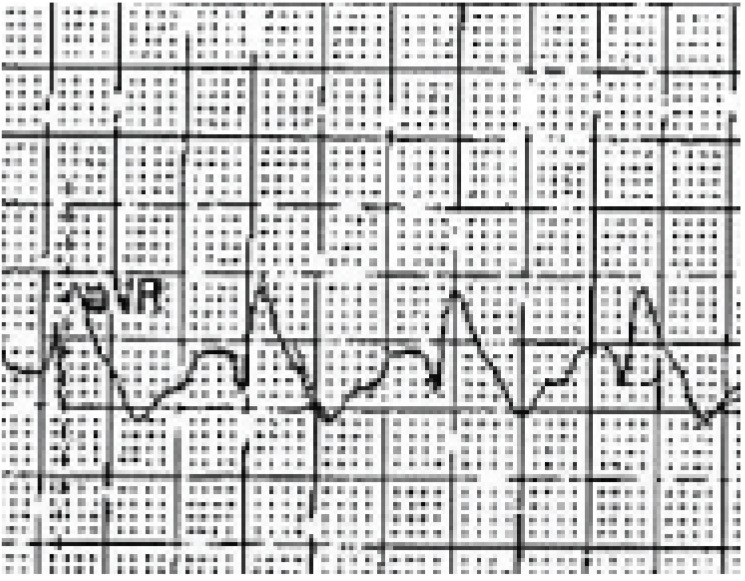
This aVR lead shows a prominent R wave, a hallmark of sodium channel blockade in TCA overdose.

**Fig. (9):**
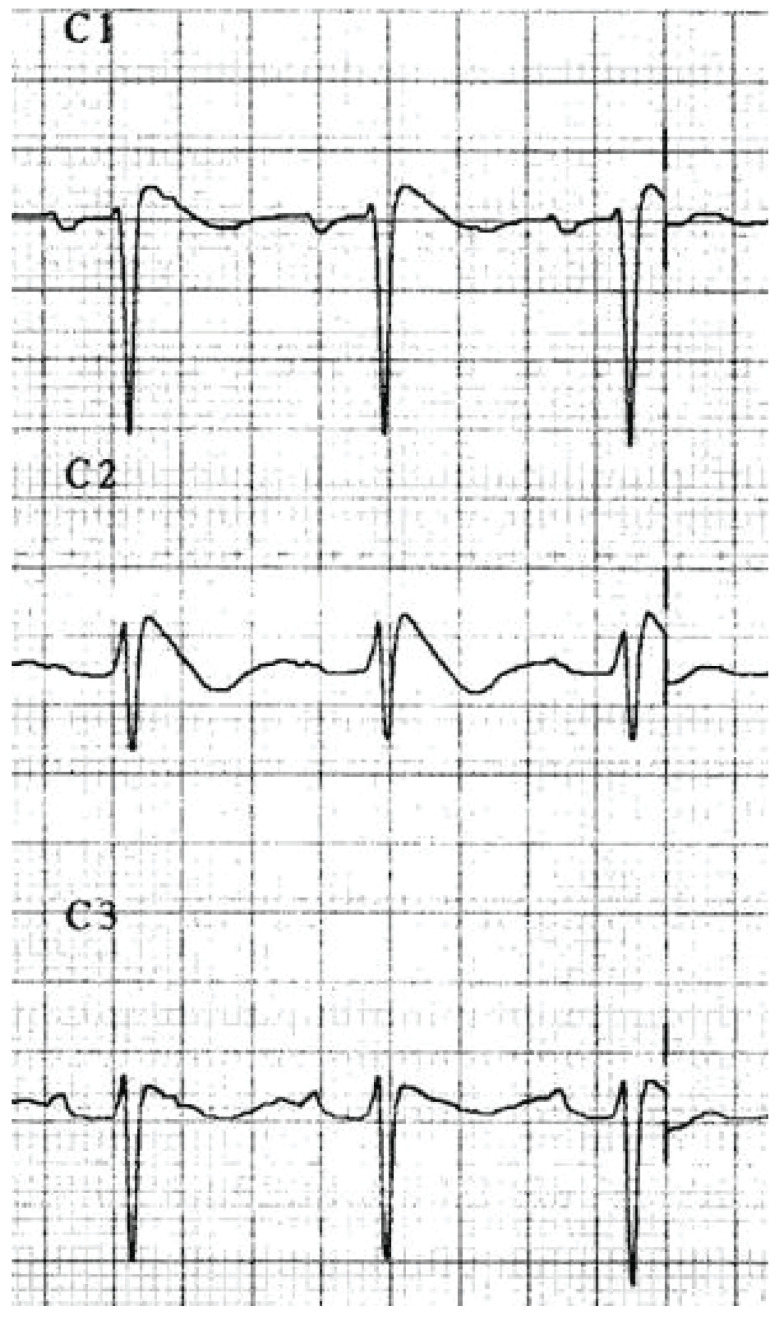
Coved ST segment elevation and negative T waves in leads V1-V3 showing a type-1 Brugada pattern.

The right–sided intraventricular conduction system is more susceptible to toxic effects of some sodium channel blockers relative to the left bundle. This phenomenon of preferential bundle branch vulnerability to drug cardiotoxicity provides some toxicology-specific ECG findings in poisoned patients. Delayed depolarization of the right ventricle can be seen in changes in the morphology of the QRS complex in aVR, which causes the presence of prominent R waves in lead AVR (Fig. **8**) along with rightward terminal 40-ms axis deviation [13, 14]. In addition, other signs of preferential right ventricular depolarization delay common to other sodium channel disturbances may include Brugada pattern (Fig. **9**) and right bundle branch block [15].

**Fig. (10):**
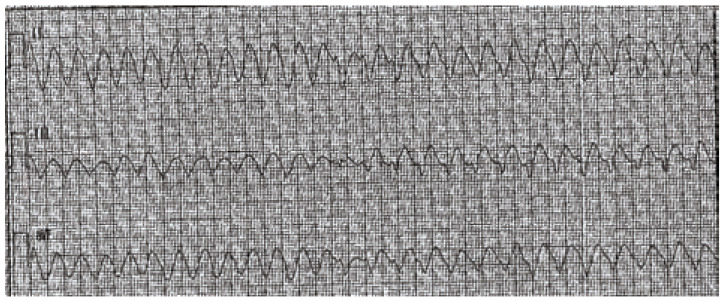
Rhythm strip demonstrating wide complex tachycardia.

As discussed above, a prolonged QRS is suggestive of sodium channel blockade. When accompanied by anticholinergic or sympathomimetic effects, the resulting rhythm may resemble ventricular tachycardia (Fig. **10**). Despite 120 ms being a standard indicator of a wide QRS for intraventricular conduction disturbance or ischemic cardiomyopathy, previously healthy, often young individuals should be expected to have shorter QRS. In fact, a prospective study published by Boehnert *et al*. in 1985 in TCA poisoned patients demonstrated that a QRS duration of under 100 ms was an indicator of good prognosis, while those with a QRS over 100 ms presented with seizures in one third of cases [16]. In the same study, a QRS complex over 160 ms was associated with ventricular dysrhythmias [16]. 

Signs of TCA poisoning and prediction of clinical toxicity can also be elucidated by examining the axis of the terminal 40 ms of the QRS complex. Its measurement can however be difficult in daily clinical practice. ECG signs of an abnormal terminal 40 ms axis are an S wave in leads I and aVL, or a prominent R wave in aVR [13]. An easily measured indicator of possible TCA toxicity is an R wave in aVR ≥ 3 mm. R in aVR has been demonstrated to predict dysrhythmia in patients with tricyclic antidepressant poisoning [14]. Clinical experience, small prospective studies and anecdotal reports support the utility of the QRS interval in the evaluation of suspected sodium channel blockade poisoning. There are however certain limitations to using an initial ECG’s QRS complex to predict outcome. A meta-analysis of prognostic indicators found the ECG to be less useful and equivalent to TCA blood concentrations [17]. This may be explained in part by the fact that inter-rater agreement and the timing of ECG recording have an influence on the value of QRS complex measurement as a tool to predict outcome and are a limitation to comparing results among various studies [18, 19]. Predicting extracardiac manifestations of toxicity such as seizures based on the ECG of patients suffering from sodium channel poisoning is conditioned by different toxic profiles of sodium channel blocking drugs and the pathophysiology of these extracardiac events [20]. 

### J wave

**Fig. (11):**
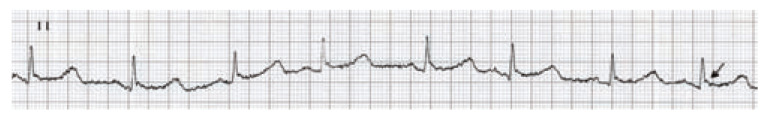
hypothermic patient's ECG strip showing bradycardia, a positive deflection at the QRS/ST junction known as an Osborn or J wave (marked by the arrow) and a long QTc interval.

The presence of a J wave, or Osborn wave may be present in patients with hypothermia [21]. This may be important in patients with depressed levels of consciousness from overdose and subsequent exposures to cold environments for prolonged time periods. (Fig. **11**) demonstrates the Osborn wave in an elderly patient with hypoglycemia due to sulfonylurea toxicity, found unconscious on her cold apartment floor.

### QT Interval

**Fig. (12):**
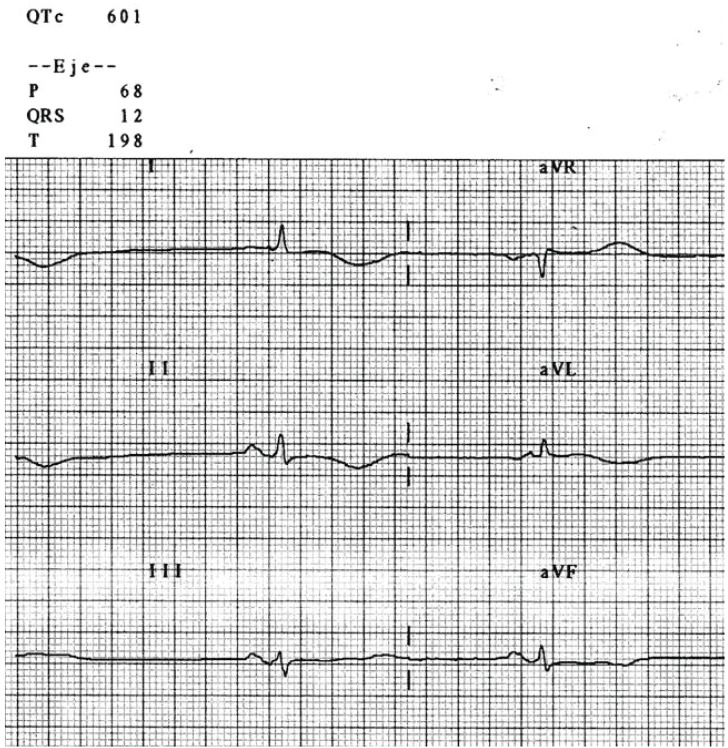
This poisoned patient's ECG shows sinus bradycardia with a severe QTc prolongation (automated calculation at 601 ms) indicating risk for TdP

The QT interval is often prolonged in overdoses involving cardiotoxicity (see Fig. **12**). Although dependent on membrane potential repolarization and especially potassium channel blockade, a prolonged QRS due to sodium or calcium channel antagonism may also prolong the QT interval. Many drugs are known to produce acquired long QT in both therapeutic dose and overdose. Several agencies keep track of the ever-growing list of drugs known to cause prolonged QT as well as TdP, including the US FDA [22] and the University of Arizona Center for Education and Research on Therapeutics [23]. For reasons that are poorly understood, many drugs known to produce potassium efflux blockade and QT prolongation have not been reported to trigger TdP. Some commonly used drugs that cause QT prolongation and TdP are summarized in Table **2**.

It is thought that drug-induced vulnerability to ventricular dysrhythmias may be detected on the ECG by examination of the QT interval duration [24,25]. QT prolongation pharmacologically occurs via three predominant mechanisms: slowed recovery from inactivation of sodium channels [26], delayed inactivation of sodium channels [27, 28], and potassium channel blockade (e.g. rapid potassium rectifier current, or I_Kr_) [29]. Detailed animal experiments show that prolongation of ventricular repolarization is a prerequisite for some ventricular dysrhythmias, particularly TdP [30]. The utility of QT interval measurement to risk stratify for prediction of adverse cardiovascular events was clearly demonstrated in pilot data from a case-control study evaluating an undifferentiated population of drug overdoses [31], but further data is needed in this area.

### Measurement 

The QT interval represents electrical depolarization / repolarization of both ventricles, and is measured from the beginning of the QRS to the end of the T wave. In poisoned patients, a lead with well recognizable T waves should be chosen for this measurement. Manual measurement can be employed for monitoring changes in serial ECG in overdose patients, but automated measurement of the QT interval is believed to be accurate and may be based on a single lead [22].

### Interpretation and Correction for Heart Rate

#### Traditional: Bazzet's Correction

Several formulas have been proposed to adjust the QT for heart rate and obtaining corrected QT (QTc). Bazzet’s formula is perhaps the most widely used and is determined by QT /√RR. A limit to define prolonged QTc is debated and it is difficult to classify those patients with a prolonged QTc at greater risk of developing TdP. In general, a QTc of over 450 ms in males and 470 ms in females is considered prolonged. A QTc greater than 500 ms is considered to be a marker for high risk of TdP. It should be noted that Bazzet’s formula overestimates QTc for tachycardic patients, so a long QTc in those patients may be more due to the correction than to an increased risk of TdP.

Results of several large epidemiologic studies have yielded conflicting results about the relationship between QTc prolongation and sudden cardiac death [32]. In patients with cardiac disease, QTc prolongation predicts myocardial injury and TdP [33,34]. Extrapolation of this data to the poisoned patient is tempting but requires further study. 

### QT Nomogram

**Fig. (13):**
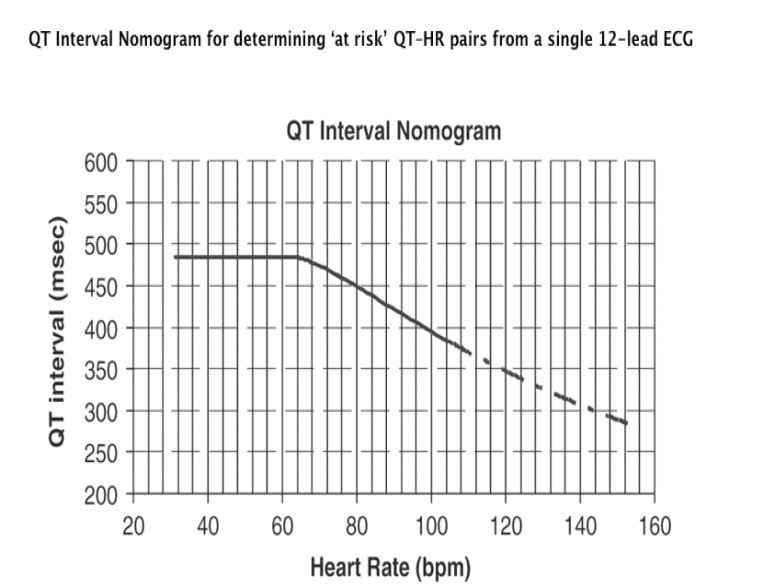
QT Interval Nomogram for determining ‘at risk’ QT–HR pairs from a single 12-lead ECG. Use: The QT interval should be measured manually on a 12-lead ECG from the beginning of the Q wave until to the end of the T wave in multiple leads (i.e. six leads including limb and chest leads and median QT calculated). The QT interval is plotted on the nomogram against the heart rate recorded on the ECG. If the point is above the line then the QT–HR is regarded ‘at risk’. Reproduced with permission from the author [35].

A QT nomogram (Fig. **13**) developed as a risk assessment tool for patients potentially at risk for TdP showed better sensitivity and specificity than traditional QTc markers in a retrospective case control study of TdP cases [35]. A subsequent retrospective case control study assessed the nomogram in antidepressant exposures cases without dysrhythmias [36]. Further investigations may validate the QT nomogram’s utility as an effective decision making aid. 

### QT Dispersion

The QT interval has long been noted to vary among the individual twelve surface leads of the ECG, but increased inhomogeneity may occur during toxic exposures that alter repolarization timing [25]. Through the development of conduction blocks and changes to the refractory periods within the atria and ventricles, increased dispersion of repolarization produces a myocardial substrate, which is vulnerable to afterdepolarizations that can “trigger” lethal dysrhythmias [34]. Toxins that increase the dispersion of repolarization typically do so non-uniformly across the myocardium [37]. This phenomenon likely relates to the varying ion channel distribution within different myocardial layers and the dissimilar effects of toxins on these ion channels. The ECG metric typically used for detection of changes to the homogeneity of repolarization is measurement of QT dispersion (QTD) [25]. Risk stratification for sudden cardiac death using QTD is a Class III recommendation of the Task Force on Sudden Cardiac Death of the European Society of Cardiology, although its use has never been adequately evaluated in toxicology studies or in drug overdose literature [38]. Pilot data from a case-control study evaluating an undifferentiated population of drug overdoses could not demonstrate utility of QTD to predict adverse cardiovascular events [31], but may need further investigation. Thus, there is insufficient data to recommend routine measurement of QTD for risk stratification in poisoning at this time. 

#### ST Segment

**Fig. (14):**
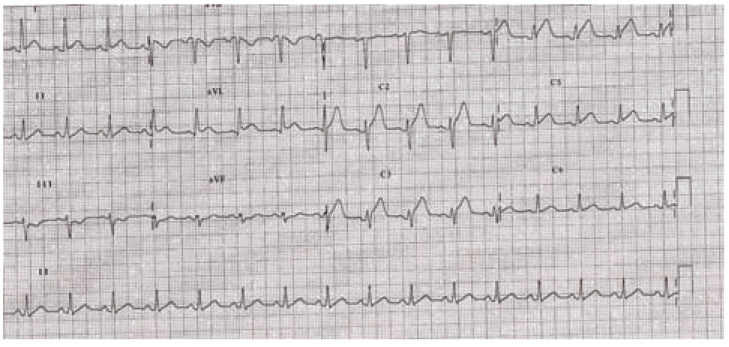
ST elevation MI in a cocaine-intoxicated chest pain patient.

ST segment depressions or elevations suggestive of ischemia or myocardial infarction may be present in overdoses involving agents known to produce vasoconstriction (e.g. cocaine, Fig. **14**). Carbon monoxide is known to produce tissue hypoxia and may cause myocardial injury [39]. By extension, any overdose associated with severe tissue hypoxia (e.g. cyanide) or hypotension (e.g. calcium channel antagonist) can produce ST segment changes due to ischemia. Pilot data from a case-control study evaluating an undifferentiated population of drug overdoses demonstrates that ischemic changes on the initial ECG predict in-hospital adverse cardiovascular events [31]. 

Brugada pattern ST segment elevation (Fig. **9**) has been described in overdoses involving sodium channel blockade. [15, 40,41] However, the predictive utility of this finding for adverse events or mortality is unclear. 

**Fig. (15):**
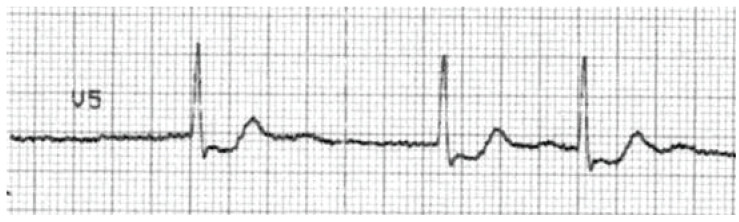
An ECG with characteristic scooping ST segment depression in a patient taking Digoxin.

Other ST segment abnormalities associated with ion disturbances may be found. A scooping ST segment (morphology often likened to Salvador Dali’s mustache), reflective of cardioactive steroid presence in myocardial tissue can occur with therapeutic or toxic levels. (see Fig. **15**). 

#### T wave

Abnormal T waves can likewise be found in patients suffering from ion disturbances or ischemia. Lithium may produce subtle changes in the T wave resembling hypokalemia. 

Terminal deflections after the T wave, or U waves, can be a reflection of early or late depolarizations in cases with prolonged repolarization.

## ECG TOXIDROME PEARLS

**Fig. (16):**
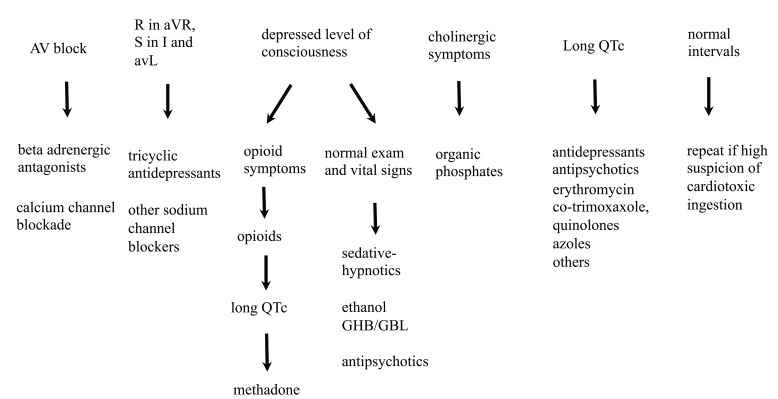
Poisoned patients presenting with a sinus rhythm should be assessed for subtle ECG signs of cardiotoxicity such as AV block, a long QTc or signs of sodium channel blockade (e.g. R in aVR). Other physical findings are included to remind clinicians to search for toxidromes and to think of poisonings that present with normal physical and ECG findings. Repeated ECGs should be performed if toxicity is suspected.

**Fig. (17):**
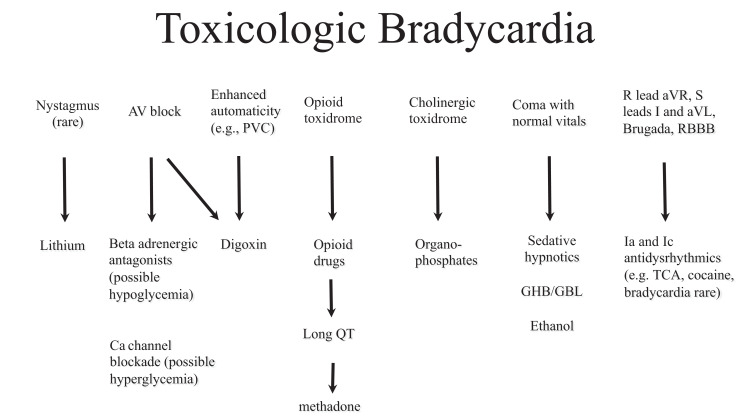
Bradycardia in poisoned patients can be assessed with the Toxicologic Bradycardia Algorithm to search for signs that may lead to suspected toxins including automaticity, hypo- or hyperglycemia or toxidrome findings. Bradycardic patients should also be assessed for sodium and potassium channel blockade (i.e. R in aVR and long QTc).

**Fig. (18):**
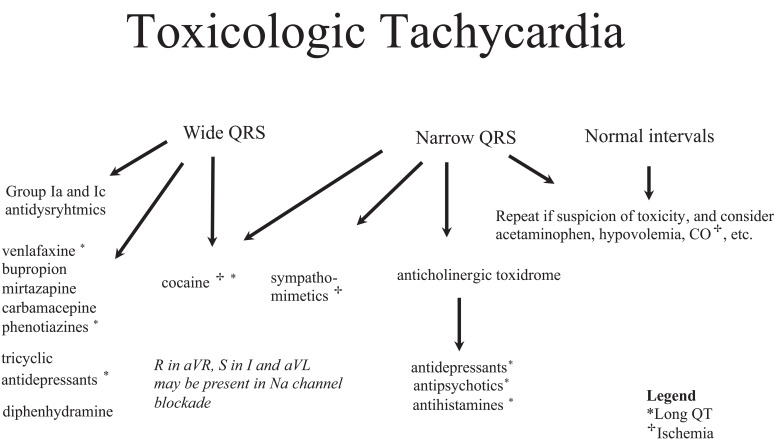
Tachycardia is a common presenting rhythm in poisoned patients. The ECG of tachycardic patients should be assessed for wide QRS complexes and other ECG signs that might suggest sodium channel blockade (e.g. R wave in aVR) as well as for QTc prolongation and ischemia. Certain physical findings can help categorize the patient in a toxidrome pattern, which together with the ECG findings may help identify toxin groups and expected effects.

Common patterns of ECG manifestations along with symptoms and physical findings in overdose patients make up what we will refer to hereafter as "ECG toxidromes". This term was originally coined to describe a common constellation of ECG and physical exam findings in hydrofluoric acid overdose [42]. We would like to present algorithms to include other scenarios commonly found in other overdose patients. Although they cannot comprehensively cover all poisonings, they illustrate the utility of the ECG in many common poisonings. To simplify the algorithms, they are divided into sinus rhythm (Fig. **16**), toxicologic bradycardia (Fig. **17**) and toxicologic tachycardia (Fig. **18**). 

## MANAGEMENT ISSUES IN CARDIOTOXICITY

### QRS Widening

Sodium bicarbonate is the initial treatment option for wide complex tachycardia and hypotension in patients with suspected drug-induced sodium channel blockade evidenced by changes in QRS morphology described above. Its mechanism of action is twofold: (a) serum alkalinization may help remove the drug (e.g. increasing tricyclic protein binding) from the receptor (i.e. sodium channel); and (b) increased extracellular sodium may partially overcome blockade of the sodium channel by the law of mass action. This is illustrated by *in vivo and in vitro *animal studies [43, 44]. Both hypertonic sodium and hyperventilation have shown positive results in TCA overdose, although a systematic review of animal and human studies found isotonic sodium bicarbonate to be the recommended first-line treatment [45].

Indications for initiation and dosing of sodium bicarbonate therapy are largely based on the experience with tricyclic poisoning [13, 14, 16,46] and are not supported by controlled clinical trials. There is considerable difference in the recommendations given by experts even when asked about alkalinization in TCA overdose, as was shown by a survey of US Poison Center Medical Directors [47]. We would recommend however a trial of sodium bicarbonate therapy for any patient with wide QRS (>100 ms) in the setting of suspected poisoning. In addition, severe dysrhythmias and wide complex tachycardia, particularly after a seizure or with concomitant metabolic acidosis, should receive a trial of sodium bicarbonate therapy. 

Conventional bolus dosing is an initial 1-2 mEq/kg bolus while the patient is attached to a cardiac monitor or ECG strip to evaluate any change to the QRS interval with therapy. The first dose is termed a “trial” of sodium bicarbonate, with the desired effect being narrowing of the QRS ideally to < 100ms. If equivocal, a repeat bolus administration may be performed. If the trial is successful, then it should be followed by an infusion of isotonic bicarbonate at twice the “maintenance” rate for intravenous fluid. (The maintenance intravenous fluids rate in adults is defined by the following equation: Rate (mL/hr) = (weight in kg) + 40). During the infusion, the serum pH should be monitored to generally avoid over-alkalinization (pH >7.55).

It should be noted that many toxins that cause sodium channel blockade might also produce a prolonged QTc (e.g. tricyclics). Thus, hypokalemia due to serum alkalinization may aggravate QT prolongation and may lead to dysrhythmia such as TdP. This should be mitigated with aggressive potassium repletion. 

The utility of hypertonic saline and hyperventilation to treat severe sodium channel blockade (e.g. tricyclics) is somewhat controversial. Hyperventilation may be an adjunctive therapy for intubated patients that require alkalinization. This may be an appropriate method of forcing respiratory alkalinization (e.g. by increasing tidal volumes) in patients who may not tolerate high volumes of isotonic bicarbonate (e.g. fluid overloaded patients). The same is true for hypertonic saline infusion for non-intubated patients. However, more study is needed to make clear recommendations regarding the safety and efficacy of hyperventilation and hypertonic saline. 

Antidysrhythmic therapy in addition to sodium bicarbonate should generally be avoided in patients with drug-induced sodium channel blockade. Lidocaine, a class IB antidysrhythmic drug, shortens the relative refractory period of the cardiac action potential, unlike class IA drugs (e.g. tricyclics). Thus, lidocaine is theoretically an attractive antidysrhythmic agent in refractory wide complex tachycardia in sodium channel blockade toxicity and is included in a review of cocaine-induced dysrhythmia [48] and recommendations for TCA poisoning [5].

Beta antagonists should be avoided or used with caution. Although they have not demonstrated increase morbility or mortality in prospective trials, they have shown adverse effects in case series and animal experimentation in cocaine intoxicated subjects and shown increased mortality in a case series and animal experimentation in TCA experience. [49-52]. Despite being a first line treatment in "regular" ACLS guidelines, amiodarone (whose effects include Beta antagonism and QTc prolongation) may not be a drug of choice in "toxicology-specific" ACLS for similar reasons and has not been studied in a poisoning scenario [5, 8,48].

### Drug-Induced QT Prolongation

As discussed above, drug-induced blockade of potassium rectifying current may lead to acquired prolonged QT syndrome and increase the risk of dysrhythmia, including TdP. (Fig. **12**) demonstrates a patient poisoned with amisulpride, an atypical antipsychotic, who developed severe QT prolongation. (Fig. **3**) demonstrates a rhythm strip from a patient who developed TdP and subsequently expired. 

Emergent treatment for patients with potassium efflux blockade who develop acute TdP or ventricular fibrillation is direct current cardioversion or defibrillation. A recent scientific statement regarding TdP prevention in the hospital setting, though not specifically referring to poisoning, recommends intravenous magnesium sulfate for patients who present with episodes of TdP or signs of impending TdP including a QTc exceeding 500 ms [53]. Other indications of impending TdP include a marked U wave, onset of ventricular ectopy and couplets, macroscopic T-wave alternans, or episodes of polymorphic ventricular tachycardia that are initiated with a short-long-short R-R cycle sequence (typically, PVC–compensatory pause–PVC) [53]. Magnesium sulfate is administered intravenously in a 2g bolus, with a repeated bolus and infusion if necessary [54]. 

Correcting electrolyte imbalances, especially hypokalemia, and hypomagnesemia as well as overdrive pacing in bradycardic patients suffering from TdP are further treatment issues to address in these patients. Overdrive pacing may be achieved chemically (e.g. isoproterenol) or electrically (e.g. transvenous pacer) in order to increase the heart rate which effectively decreases the QTc and thus the risk of TdP. Ideally overdrive pacing should be carried out in consultation with a cardiologist. 

### Drug-Induced Bradycardia

Bradycardia as a guiding symptom in toxicology is limited by the fact that many end-stage poisonings may present with severe illness including a slow heart rate, often with a wide QRS. However, those drugs that have a direct cardiotoxic effect and slow the heart rate as a central element of their mechanism of action can be expected to present classic toxidromes in overdose, with aggressive treatment strategies tailored based on mechanism of action. Some physical or ECG findings as shown in the Toxicologic Bradycardia Algorithm (see Fig. **17**) in unknown ingestions may help initial evaluation and monitoring. 

Calcium channel blockade and Beta adrenergic antagonism share similar intracellular effects at the myocardium and share some treatment targets and therapies. Sodium/ Potassium ATPase blockade has a specific treatment regimen (see below). 

### Drug-Induced Calcium Channel Blockade

Calcium channel blocking agents (especially cardiac acting verapamil and diltiazem) are known to produce severe cardiotoxicity including myocardial depression, hypotension, complete AV block, and asystole. Airway and respiratory issues will require assessment and intervention in timely fashion. Circulatory disturbances may be due to peripheral vasodilatation, myocardial conduction disturbance, depressed inotropy, or any combination thereof. 

Calcium channel blocker overdose may produce severe hyperglycemia due to reduced insulin release in the pancreas [55, 56]. Thus patients who present with bradycardia and hyperglycemia should raise suspicion of calcium channel blocker overdose until proven otherwise and should be managed appropriately.

Standard ACLS recommendations for bradycardia and hypotension (e.g. atropine, fluid resuscitation and vasopressors) may be applied with the caveat that direct-acting vasopressors (e.g. norepinephrine) are probably a more rational choice than indirect vasopressors (e.g. dopamine). Poor results can be expected with conventional treatments in severely poisoned patients and antidotal therapy is expected to be much more effective. At a minimum, these patients should receive supportive care and monitoring in an appropriate unit. 

Specific treatment for calcium channel blockade includes calcium salt infusion (1-5 g of calcium chloride or gluconate in adult patients, to be repeated every 15 minutes until recovery or hypercalcemia). Since only mildly symptomatic patients can be expected to respond to calcium, other treatment modalities should be simultaneously prepared in severe poisonings. Initial therapy in severely poisoned patients should also include high dose insulin and dextrose therapy [56, 57]. The proper dose is 1 UI/kg followed by 0.1 UI/kg/hr insulin, 25-50g 50% dextrose in adults initially, followed by 0.5 g/kg/hr titrated to blood glucose levels [55]. Further treatment options that have shown some positive effect include glucagon and phosphodiesterase inhibitors. Initial experience with lipid emulsion therapy as a last resort resuscitative measure has shown encouraging results (see below).

### Drug Induced Beta Blockade

ECG manifestations resulting from Beta adrenergic antagonism toxicity are sinus bradycardia and dysfunction with varying degrees of AV-block, as well as junctional escape rhythms (see Fig. **7**). Some Beta antagonists also demonstrate sodium channel antagonism (e.g. propranolol) while others produce potassium efflux antagonism (e.g. sotalol). In addition, Beta adrenergic antagonists may rarely produce certain clinical findings such as hypoglycemia and bronchospasm that con serve as clinical pearls to the nature of the ingestion. Propranolol and sotalol exposures, which may present with sodium channel blockade or a long QTc and TdP, respectively, should be assessed for ECG manifestations of these phenomena.

Initial treatment addressing airway, breathing are shared with calcium channel blockade. Atropine can be administered for bradycardia, and fluids for hypotension. Glucagon is the next line of treatment in patients who do not respond to fluids (50 to 150 mcg/kg bolus followed by an infusion of 1 to 5 mg/hr) and should probably precede catecholamine use. If catecholamines are required, treatment recommended for calcium channel blockade poisoning (calcium salts, high dose insulin/euglycemia) should be prescribed [55]. There may also be a role for lipid rescue therapy in severely poisoned patients with Beta adrenergic antagonists (see below).

### Digoxin and Other Cardioactive Steroid Toxicity

**Fig. (19):**
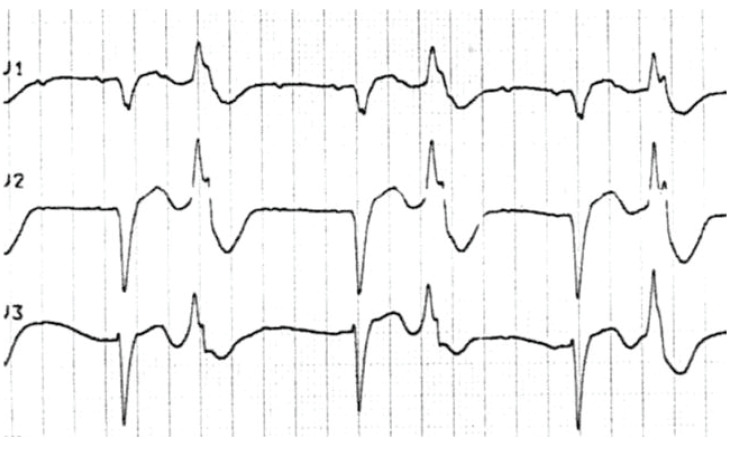
Enhanced automaticity is seen in this ECG of a patient with supra-therapeutic digoxin levels and bigeminy

**Fig. (20):**
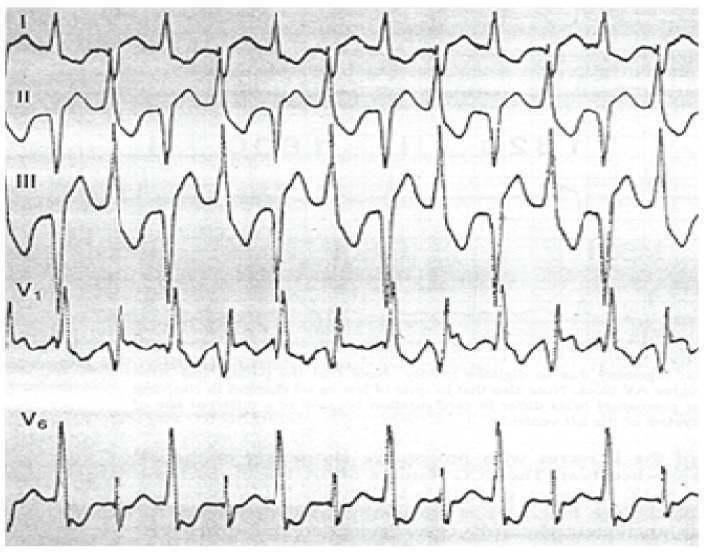
This ECG shows an alternating left and right axis polarity, relatively narrow ventricular tachycardia, known as bidirectional ventricular tachycardia and practically exclusive to cardioactive steroid poisoning

In the setting of cardioactive steroid toxicity, the cardiac manifestations of supra-therapeutic concentrations cause enhanced automaticity produced by increased intracellular calcium and depression of SA and AV conduction. The resulting ECG changes can be varied and include prolonged PR in patients with a prior sinus rhythm, any degree of AV block as well as increased irritability (see Fig. **19**) which can manifest as anything from premature ventricular contractions to ventricular fibrillation. Bidirectional ventricular tachycardia is a rare dysrhythmia found almost exclusively in cardioactive steroid toxicity (see Fig. **20**).

Noncardiac symptoms of toxicity include gastrointestinal disturbances, altered consciousness and visual disturbances and are reviewed extensively elsewhere [58].

Cardioactive steroids share toxic effects and treatments, though digoxin is the most common agent in clinical use and is the reference for treatment recommendations. Digoxin toxicity can present as acute ingestions in patients not previously taking digoxin, or as chronic toxicity in patients on digoxin therapy that have excessive dosing or reduced elimination (i.e. renal failure). ECG changes can be expected in both to include signs of myocardial impregnation with digoxin, AV conduction disturbances and ectopy. 

Besides cardiovascular effects, acute toxicity presents with gastrointestinal symptoms and in severe cases with hyperkalemia, a marker of severity and another factor in the decision for specific treatment. Chronic toxicity may present with altered mental status, gastrointestinal and visual disturbances in addition to classic ECG changes (e.g. "digitalis-effect" as in Fig. **15**). Abnormalities in the baseline ECG of patients receiving digoxin therapy will be the substrate for further alterations and should be considered when evaluating chronic digoxin toxicity.

Mild digoxin toxicity (i.e. mild symptoms without serious ECG changes or dysrhythmia) may be treated with supportive care and observation with cardiac monitoring. Specific antidotal therapy is warranted for severe poisoning and indications for digoxin-specific Fab are summarized in Table **3**. There are several different commercially available preparations of digoxin-specific Fab and clinicians should consult the preparation available at their institution. For the sake of simplicity, we include recommendations in number of vials (which reflect the 0.5 mg digoxin binding ability of 38 or 40 mg vials). The dosing recommendations are based on an estimated total body load of digoxin and a multicenter trial that established its efficacy [59]. When the serum concentration is unknown, or will be delayed, empiric dosing is based on estimation for the average requirements for an acute or chronic ingestion [58]. A proposal for decreased dosing based on pharmacokinetic principles of digoxin poisoning in a review of digoxin specific antibody fragment treatment suggests that half of the calculated dose should be more appropriate and followed by repeat doses if necessary [60], but has not been prospectively evaluated.

Successful management of cardioactive steroid toxicity from toxins other than digoxin (i.e. oleander, squill and toad venom) has been reported and should follow an empiric treatment regime and further dosing according to response [61]. 

Digoxin toxicity does not respond well to electrical pacing, which has been associated with adverse outcomes (pacing induced dysrhythmia) and should only be performed after Fab therapy has failed as a last resort in the patient with cardiovascular collapse [62]. 

###  Normal Sinus Rhythm

The Sinus Rhythm Algorithm (see Fig. **16**) shows some common clinical pearls that may be found in the poisoned patient. Subtle signs of sodium and potassium channel disturbances as well as AV conduction changes should be monitored on serial ECG to discover evidence of cardiotoxicity. 

A 6 to 8 hour period of observation in patients with a normal sinus rhythm without other signs of cardiotoxicity and otherwise normal physical findings may be sufficient except in cases exposed to sustained release preparations or drugs with known delayed toxicity (e.g. citalopram) [63]. A reasonable period of observation may be 24 hours in these patients if their ECG remains normal, although the safety of this recommendation has not been studied.

### Sinus Tachycardia

Sinus tachycardia is a common presenting rhythm in clinical toxicology. Thus other signs of cardiotoxicity or toxidrome findings as shown in the Toxicologic Tachycardia Algorithm (see Fig. **18**) can be helpful to guide evaluation. Suspected exposures to cardiotoxic substances should be monitored and signs of sodium channel blockade and QTc abnormalities scrutinized in serial ECGs. If hypovolemia is considered to be a factor, fluid challenge may be efficacious. 

### Prediction of In-Hospital Prognosis Using the ECG 

**Fig. (21):**
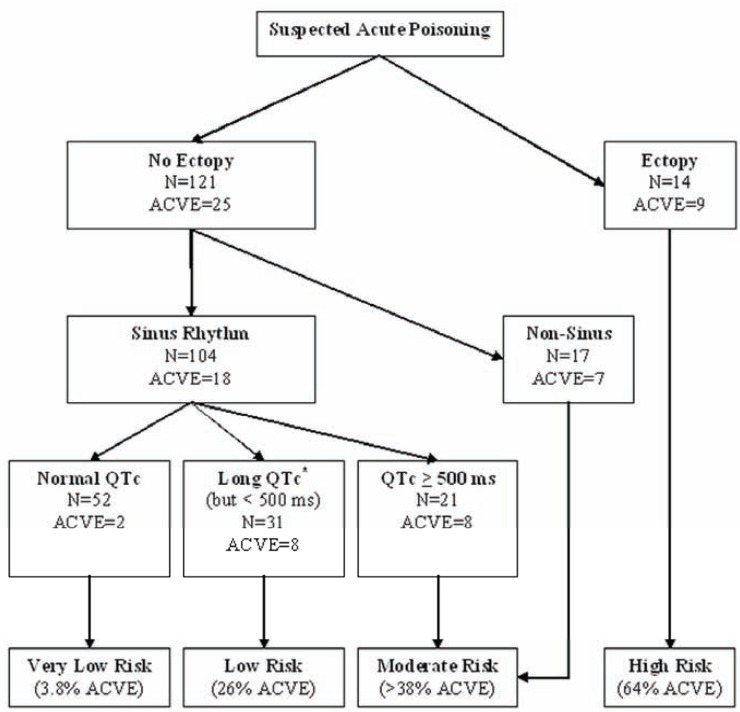
Risk stratification scheme based on a case control study of an undifferentiated population of patients with diverse poisonings [31]. Categories were achieved by recursive partitioning based on ECG elements associated with risk of ACVE (see text for definition), *Long QTc was defined as ≥ 470 ms in females and ≥ 450 ms in males. ms = milliseconds; QTc = corrected QT interval. Reproduced with permission.

ECG toxidromes may not be limited to just helping clinicians tailor management and antidotal therapies. It is likely that data from the ECG may predict in-hospital prognosis for patients with undifferentiated exposures. In a case-control study evaluating the initial ECG of patients with acute drug overdose, findings associated with adverse cardiovascular events (ACVE, defined by the following composite endpoint: (a) myocardial injury, (b) ventricular dysrhythmia, (c) shock, or (d) cardiac arrest) included the following: ischemic changes, QT prolongation, ectopy, and non-sinus rhythm [31]. An algorithm suggested by the authors to risk stratify acute overdose in-hospital prognosis based on the initial ECG is reproduced in (Fig. **21**). 

## ACUTE CORONARY SYNDROME AND COCAINE

Specific management and treatment recommendations have been made for cocaine-associated chest pain and myocardial infarction (MI) by the American Heart Association which include an early invasive strategy for MI, the role for benzodiazepines and aspirin in these patients and the proscription of beta adrenergic blockade [64]. A brief observation period for cocaine-associated chest pain is also recommended, based on a study by Weber and associates [65].

### Cardiac Arrest

Poisoned patients suffering from cardiac arrest should be treated according to ACLS guidelines with consideration of toxicology-specific antidotes as adjunctive therapy. For example, specific treatments such as early sodium bicarbonate (e.g. TCA), digoxin-specific Fab therapy, high dose insulin euglycemia (e.g. calcium channel antagonist) and lipid resuscitation therapy (e.g. bupivacaine) may be indicated. Therapeutic hypothermia post cardiac arrest should be considered if there is return of spontaneous circulation. 

Some experience exists with extracorporeal life support (ECLS) in patients severely poisoned with refractory cardiovascular collapse [66, 67]. Unfortunately, this approach is not readily available in many hospital settings. No definitive recommendation can be made based on the limited data in the literature, but the French ICU experience is encouraging. In select cases, patients have recovered without sequelae from drug-related cardiac arrest following implementation of ECLS resuscitation measures. Thus, ECLS may be considered in refractory cardiovascular collapse, where available and in consultation with a medical toxicologist, an intensivist, and a cardiothoracic surgeon.

## LIPID RESUSCITATION THERAPY

Intravenous lipid emulsion (ILE), composed of triglycerides, phospholipids and glycerol, was approved by the FDA in 1972 for use in parenteral nutrition. Lipid resuscitation therapy (LRT) refers to the administration of ILE with the intent of reducing the clinical manifestations of toxicity from local anesthetic (e.g. bupivacaine) overdose or from lipophilic medication overdose. LRT has gained widespread acceptance to treat local anesthetic toxicity [68]. Animal studies and several case reports have described benefits of LRT to treat cardiovascular collapse due to overdose of lipophilic drugs [69,70]. IV preparations of ILE (trade name Intralipid®) are available and consist of 10%, 20% and 30% concentrations. However, because high doses of ILE are required for LRT and adverse events related to high-dose ILE administration are poorly studied, at this time LRT is considered a rescue intervention of last resort. 

The decision to use LRT instead of, or in conjunction with, other therapies that have been anecdotally reported to be effective (e.g. hyperinsulinemia euglycemia therapy), is to be based on the clinical judgment of the treating physician. Due to rapidly developing experience, when possible, it is recommended that such therapies be administered with consultation from a medical toxicologist or guidance from the regional poison control center. Guidelines for this use recommend an initial 1.5 ml/kg bolus followed by a 0.25 ml/kg/min perfusion and repeated boluses and twice the perfusion rate if necessary [68]. Systematic review of lipid emulsion as an antidote for cardiotoxicity resulting in cardiac arrest demonstrates animal experiments and isolated case reports of its use only [69,70]. Some authors also recommend LRT to resuscitate cardiovascular collapse due to Beta adrenergic antagonists, verapamil, tricyclic antidepressants, bupropion/lamotrigine and sertraline/quetiapine exposures [69], while Brent suggests that any patient suffering from cardiac arrest not responding to standard treatment after cardiotoxic drug overdose should receive an expeditious trial of LRT [71]. A recent article highlights the difficulties in evaluating results in these critically ill patients, including methodological and ethical issues, and that further indications will depend on the availability of rigorous scientific evidence [72].

## Figures and Tables

**Table 1.** Sodium Channel Blocking Drugs According to Pharmacological Classification

Table 1. Commonly used drugs that cause sodium channel blockade.

Anticonvulsants
Carbamazepine
Antidysrhythmics
Group IA and IC
Group II (Propranolol)
Group IV (Diltiazem and Verapamil)
Antihistamines
Diphenhydramine
Antimalarial drugs
Chloroquine
Hydroxychloroquine
Quinine
Antipsychotics
Phenothiazines
Drugs of abuse
Cocaine
Opioids
Propoxyphene
Other antidepressants
Bupropion
Mirtazapine
Venlafaxine
Tricyclic Antidepressants
Amitriptyline
Desipramine
Doxepin
Imipramine
Nortriptyline

**Table 2.** A list of selected drugs that produce potassium efflux blockade. * Denotes drug with high risk of TdP. For a complete list of QT drugs see the Arizona CERT database at www.qtdrugs.org

**Table 2.** Commonly used drugs that cause potassium efflux blockade.		

Albuterol	Erythromycin*	Phentermine
Amantadine	Escitalopram	Phenylephrine
Amiodarone*	Fenfluramine	Phenylpropanolamine
Amitriptyline	Flecainide	Procainamide*
Dextroamphetamine	Fluconazole	Protriptyline
Amphetamine	Fluoxetine	Pseudoephedrine
Arsenic trioxide*	Fosphenytoin	Quetiapine
Astemizole *	Gatifloxacin	Quinidine*
Atomoxetine	Gemifloxacin	Risperidone
Azithromycin	Haloperidol*	Ritodrine
Chloral hydrate	Ibutilide*	Ritonavir
Chloroquine*	Imipramine	Salmeterol
Chlorpromazine*	Isoproterenol	Sertindole
Ciprofloxacin	Itraconazole	Sertraline
Cisapride*	Ketoconazole	Sotalol*
Citalopram	Levalbuterol	Sparfloxacin*
Clarithromycin*	Levofloxacin	Tacrolimus
Clomipramine	Lithium	Tamoxifen
Clozapine	Methadone *	Telithromycin
Cocaine	Methylphenidate	Terbutaline
Desipramine	Mexiletine	Terfenadine*
Dexmethylphenidate	Midodrine	Thioridazine*
Diphenhydramine	Moxifloxacin	Tizanidine
Dobutamine	Nicardipine	Trazodone
Domperidone *	Norepinephrine	Trimethoprim-Sulfa
Dopamine	Nortriptyline	Trimipramine
Doxepin	Ofloxacin	Vardenafil
Droperidol *	Ondansetron	Venlafaxine
Ephedrine	Paroxetine	Ziprasidone
Epinephrine	Pentamidine*	

**Table 3.** Common indications and dosing recommendations for Digoxin-specific Fab. The dosing schedule varies according to the specific clinical scenario and whether the ingested amount or blood serum levels are known. Dosing is based on 38 or 40 mg vials. Adapted from Goldfrank's Toxicologic Emergencies, Ninth Edition [58]

**Table 3**. Indications and dosing for digoxin-specific Fab.

Indications: Symptomatic Dysrhythmia (e.g. bradydysrhytmias, ventricular dysrhythmias) Acute poisoning with serum potassium of over 5 mEq/L, Ingestion of over 4 mg in a child (0.1 mg/kg) or 10 mg in an adult, 4. A serum digoxin level of over 10 ng/mL in steady state measurement, or of over 15 ng/mL at any time.
Dosing: Unknown acute ingestion, 10 vials IV and repeated if necessary Life threatening toxicity, 20 vials Known amount, number of vials = digoxin ingested in mg multiplied by 0.8/ 0.5 mg bound per vial. For chronic ingestions: number of vials = (serum digoxin concentration in ng/mL) x (patient weight in kg)/100 rounded up to nearest integer.
